# Recommendations on Off-Label Drug Use in Pediatric Guidelines

**DOI:** 10.3389/fphar.2022.892574

**Published:** 2022-06-09

**Authors:** Min Meng, Qi Zhou, Wenjuan Lei, Min Tian, Ping Wang, Yunlan Liu, Yajia Sun, Yaolong Chen, Qiu Li

**Affiliations:** ^1^ Department of Chevidence Lab Child & Adolescent Health, Children’s Hospital of Chongqing Medical University, Chongqing, China; ^2^ Ministry of Education Key Laboratory of Child Development and Disorders, National Clinical Research Center for Child Health and Disorders, China International Science and Technology Cooperation Base of Child Development and Critical Disorders, Children’s Hospital of Chongqing Medical University, Chongqing, China; ^3^ Chongqing Key Laboratory of Pediatrics, Chongqing, China; ^4^ Department of Pharmacy, Gansu Provincial Hospital, Lanzhou, China; ^5^ Institute of Health Data Science, Lanzhou University, Lanzhou, China; ^6^ Evidence-Based Medicine Center, School of Basic Medical Sciences, Lanzhou University, Lanzhou, China; ^7^ School of Public Health, Lanzhou University, Lanzhou, China; ^8^Research Unit of Evidence-Based Evaluation and Guidelines, Chinese Academy of Medical Sciences (2021RU017), School of Basic Medical Sciences, Lanzhou University, Lanzhou, China; ^9^WHO Collaborating Centre for Guideline Implementation and Knowledge Translation, Lanzhou, China; ^10^GRADE Center, Lanzhou University, Lanzhou, China; ^11^Department of Nephrology, Children's Hospital of Chongqing Medical University, Chongqing, China

**Keywords:** pediatric guidelines, recommendations, off-label use of drugs, evidence-based medicine, children

## Abstract

**Objective:** To systematically analyze the supporting evidence, drug information, and the type of off-label drug use in recommendations on off-label drug use in pediatric guidelines.

**Methods:** A cross-sectional study was performed by systematic search through MEDLINE (*via* PubMed) and Embase databases to identify literature published from 1 January 2018, to 31 December 2020. Only pediatric clinical practice guidelines that included recommendations on off-label use of drugs were included. We present descriptive information on the sources of the included guidelines, country, publication year, evidence grading system used, details on the types of off-label drug use, and the types of studies used as references to support the recommendations.

**Results:** A total of 66 pediatric guidelines with 605 recommendations were included. Eighty-seven (14.4%) recommendations did not cite any references; and the remaining 518 recommendations were supported by 2,240 references (mean 4.3 references/recommendation). The most common types of studies cited were pediatric RCTs (*n* = 314, 14.0%), pediatric case series studies (*n* = 260, 11.6%), and reviews (*n* = 255, 11.4%). Twenty-one percent (*n* = 470) of the references were studies on adults. One hundred and forty (23.1%) recommendations were graded using the Grading of Recommendations, Assessments, Development, and Evaluations (GRADE) system, of which 37 (26.4%) were graded as strong but supported with only C or D level of evidence. The most commonly reported type of information in the recommendations was indication (n = 499, 82.5%). The most commonly addressed type of off-label drug use in the 523 positive recommendations was unapproved population (*n* = 255, 48.8%). Sixty-nine (11.4%) recommendations explicitly reported the drug use as off-label.

**Conclusion:** Children may be exposed to medical risks due to gaps in reporting and evidence of off-label drug use recommendations in pediatric guidelines.

## Introduction

Off-label use of drugs is highly common in pediatrics (Schrier et al., 2020; Sweileh, 2021) due to delays in updating drug instructions and difficulties in conducting clinical trials (Hudgins et al., 2018; Hwang et al., 2018; Wu et al., 2019; Carmack et al., 2020). The prevalence of pediatric off-label drug prescriptions has been estimated to range from 3.2% to 95% overall, 26%–95% in neonates (Allen et al., 2018; Balan et al., 2018), 2.7%–51.2% in outpatients, and 9.0%–79.0% in inpatients (Li et al., 2016; Zhou Y. et al., 2021). However, sometimes off-label use of drugs could be inappropriate or without proven therapeutic benefit. Medical decisions involving off-label drug use should thus be based on the existing evidence (Frattarelli et al., 2014; Ito, 2017) to avoid irrational medication use. Clinical Practice Guidelines (CPGs) that aim to collect, grade and summarize the latest available evidence are the best option for mitigating the risk of irrational pharmaceutical use and the liability associated with the off-label use of drugs (guidelines., 2011).

At present, there are gaps in the reporting and evidence of off-label drug recommendations in pediatric guidelines. The lack of evidence for pediatric off-label use of drugs raises concerns about efficacy and safety. Some guidelines did not mention that the recommended treatment was off-label, potentially complicating clinical practice (Kochanek et al., 2019). In addition, there is often substantial variation in the information reported between different guidelines that recommend the same drug for off-label use (Zhou P. et al., 2021). Indications, dosage, cautions, medication regimen, side effects, pharmacological mechanisms, and drug interactions are all included in the guidelines of specific drugs. However, guidelines not focusing on any specific drug are usually less comprehensive in reporting drug related information. Moreover, there are more adverse drug reactions associated with off-label prescribing that are not supported by good evidence (Eguale et al., 2016). As a result, there is a need to better clarify the current evidence for pediatric off-label drug use in pediatric guidelines to assist in the judicious use of drugs.

This study focuses on recommendations on off-label drug use in pediatric guidelines published in the last 3 years, with the aim to demonstrate the variability in reporting recommendations, present information on drugs recommended for off-label use for different disease categories, and analyze the evidence on off-label drug use in pediatrics. Our study will provide a reference for guideline developers to present recommendations on off-label use of drugs and for pediatricians to guide the off-label administration of drugs.

## Methods

### Study Design

A cross-sectional study was conducted.

### Search Strategy

We performed a systematic search through MEDLINE (*via* PubMed) and Embase databases to identify literature published from 1 January 2018, to 31 December 2020, with the terms “infant,” “newborn*,” “adolescent,” “child*,” “pediatric*,” “guideline*,” and “recommendation*” (Supplementary Table S1).

### Inclusion and Exclusion Criteria

We included articles that met the following criteria: 1) the article was a guideline published in an academic journal between 2018 and 2020; 2) the guideline addressed exclusively pediatric clinical practice; and 3) the guideline contained recommendations on off-label use of drugs. Duplicates, guidelines that included adults, translated guidelines, executive summaries, or articles published in languages other than English were excluded.

### Study Selection

Retrieved records were exported into Endnote X9 (version 9.3.1). Six investigators were divided into three groups (Group 1: Min Meng & Ping Wang, Group 2: Yunlan Liu & Wenjuan Lei, and Group 3: Min Tian & Yajia Sun). The identified records were divided between the three groups. In all groups both investigators independently screened first the titles and abstracts of the identified records, and then the full texts of the potentially eligible articles. Disagreements were solved by consensus or consultation with the senior investigators.

### Data Extraction

Two pharmacists with experience in clinical pharmacy independently extracted the information in the guidelines containing recommendations related to the off-label use of drugs. For pediatric guidelines with clear recommendations, the information on the off-label drug was further analyzed. The extracted information included: 1) basic information: publisher (organization), publication time, target population, disease category, disease (Pocai, 2019) (ICD-11 classification), number and content of recommendations, number of positive and negative recommendations (see definition below), used evidence grading systems, and conflict of interests (Supplementary Table S2); 2) Information on off-label drug use: content of the recommendations, name of the drug (or drug class, depending on whether a specific drug or a class of drugs is mentioned in the recommendation), details of the drug administration (indication, route of administration, dose, precise dosage, course of treatment, precautions, adverse reactions, contraindications, others), types of off-label drug use (see categorization in the next paragraph), types of cited literature, content of the evidence, and the level of recommendation according to the GRADE grading system (Supplementary Table S3) (Atkins et al., 2004a).

A negative recommendation was defined as a recommendation using unambiguously negative terms, such as “no,” “against,” “avoid,” or “contraindication.” A suggestion was considered positive if it contains both positive and negative descriptions. The types of off-label use were categorized into the following groups (Aronson and Ferner, 2017): 1) unapproved indication: the drug was recommended for a condition not mentioned in the drug instructions; 2) unapproved age: the drug was recommended for children outside the age range specified in the instructions; 3) unapproved population group: the drug was approved for the indication in adults but not in children; 4) unapproved route of administration; 5) unapproved dosage or frequency: dose and frequency inconsistent with the description in the instructions; or 6): unlicensed: the drug or hospital preparation was not approved or was withdrawn, by the US Food and Drug Administration (FDA). The FDA instructions and the Micromedex pharmaceutical information system were used to determine the drug class and type of off-label use (https://www.micromedexsolutions.com/). If a pharmaceutical could be classified into multiple drug classes, we determined the most appropriate drug class based on the ailment addressed in the guidelines (Supplementary Table S3).

Two investigators independently extracted the data and conducted two pilot rounds to improve consistency. Discrepancies were resolved through consultation with the senior investigators.

### Data Analysis

A descriptive statistical analysis of the included studies was performed, which presents the characteristics of the guidelines and information on off-label drugs. The distribution of reference types for off-label drug recommendations, drug ranking (highlighted drugs are on the WHO Model List of Essential Medicines for Children - 8th list, 2021 (WHO, 2021b)), and drug classes were also studied descriptively. Excel 2021 was used to collect and analyze the data (version 16.54).

## Results

### Characteristics of Included Guidelines

**FIGURE 1:**
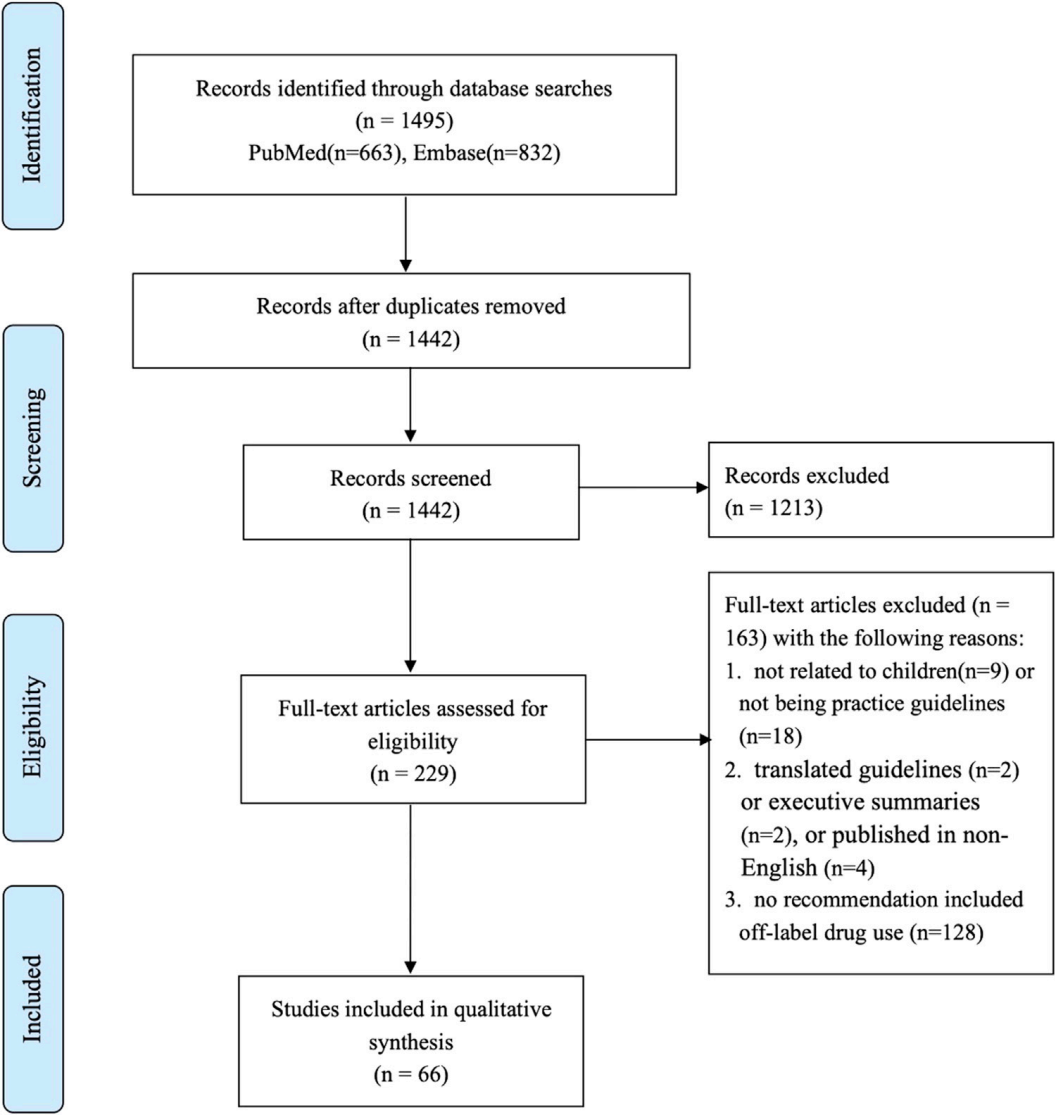
Guideline screening process.

We identified 1,465 articles through the electronic database searches, and 66 guidelines (containing 605 recommendations) were finally included after the title/abstract and full-text screening. The full process of the literature search is shown in Figure 1.

The first authors of the 66 guidelines were from 18 countries, with nearly one-third from the United States (*n* = 19, 28.8%), and more than 80% (*n* = 58, 87.9%) from high-income countries. A grading system for evidence was adopted in 42 (63.6%) guidelines, conflicts of interest was declared in 19 (28.8%) guidelines, with 9 (13.6%) guidelines may had commercial involvement in the recommendation of off-label use, and 56 (84.8%) guidelines were produced by professional societies or organizations.

Endocrine, nutritional, or metabolic disorders (n = 7, 10.6%), and immune system diseases (n = 7, 10.6%) were the most frequently addressed disease systems. The number of recommendations could not be counted in 20 (30.3%) guidelines because of the ambiguousness of the recommendations. Forty-six (69.7%) guidelines had clear recommendations, of which twelve only discussed drugs. Table 1 lists the characteristics of the included guidelines.

**TABLE 1:** Characteristics of the included guidelines.

Characteristic		Number of articles	Percentage (%)
First author’s country	United States	19	28.8
	United Kingdom	8	12.1
	Australia	5	7.6
	Canada	5	7.6
	France	5	7.6
	Italy	5	7.6
	Other^a^	19	28.7
Publication year	2018	29	43.9
	2019	19	28.8
	2020	18	27.3
Disease system	Endocrine, nutritional or metabolic diseases	7	10.6
	Immune system diseases	7	10.6
	Respiratory diseases	6	9.1
	Digestive system diseases	6	9.1
	Diseases of the blood or blood-forming organs	5	7.6
	Other^b^	35	53.0
Evidence grading system	Reported	42	65.2
	Not reported	24	36.4
Conflicts of interest	Yes	19	28.8
	No	29	43.9
	Without reported	18	27.3
Developer	Professional associations and organizations	56	84.8
	Group of individuals	4	6.1
	Not reported	6	9.1
Clarity of recommendations^c^	Clear	47	71.2
	Unclear	19	28.8
Proportion of recommendations on drug use^d^	0%~49.9%	28	60.9
	50.0%~99.9%	15	32.6
	100%	3	6.5
Proportion of drug use recommendations on off-label use^e^	0%~49.9%	14	30.4
	50.0%~99.9%	20	43.5
	100%	12	26.1

a India, Germany, Israel, South Africa, Saudi Arabia, Austria, Japan, China, Turkey, Brazil, Poland, and South Korea.

b Musculoskeletal or connective tissue diseases; signs, symptoms, or clinical findings; ear or mastoid disease; skin diseases; visual system disorders; certain infectious or parasitic diseases; circulatory system diseases; genitourinary diseases; injury, poisoning, or some other external cause; neurological diseases; tumors, and others that could not be classified.

c The recommendations were clear if they were explicitly highlighted (e.g., in bold face) or stated individually, and unclear if they were narrative and embedded in paragraphs throughout the guidelines.

d The number of drug-use recommendations divided by the total number of recommendations.

e The number of off-label drug recommendations divided by the number of recommendations on drug use.

### Types of References for Recommendations on Off-Label Drug Use

**FIGURE 2:**
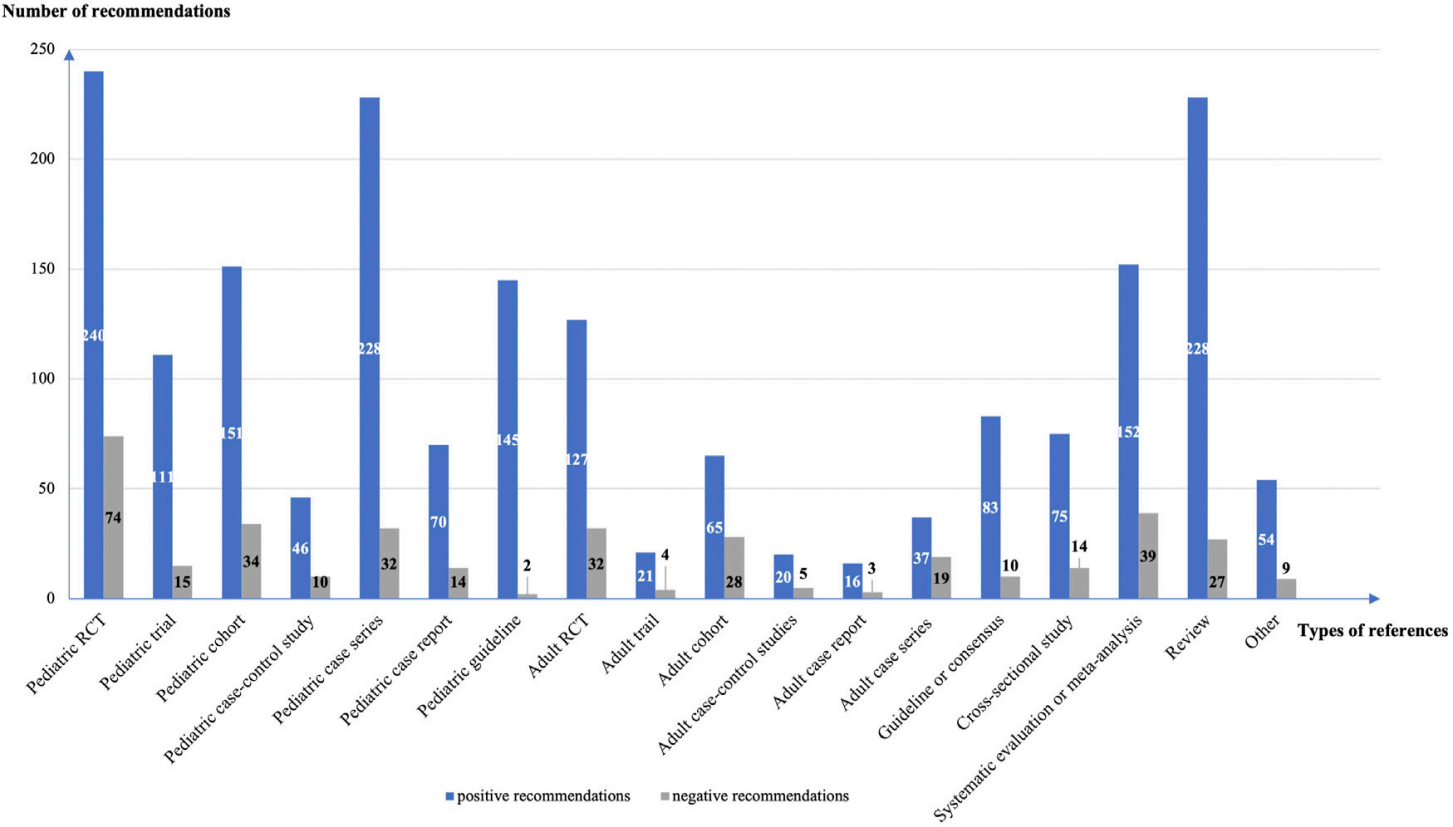
The types of references cited to support the recommendations.

The guidelines contained a total of 605 recommendations on off-label drug use, including 523 positive (86.4%) and 82 negative (13.6%) recommendations. Eighty-seven (14.4%) recommendations had no references. We analyzed 1869 references for the 441 positive recommendations with references, and 371 references for 77 negative recommendations with references. The mean number of references per recommendation was 4.3 (4.2 for positive recommendations and 4.8 for negative recommendations). Pediatric randomized controlled trials (*n* = 314, 14.0%), pediatric case series (*n* = 260, 11.6%), and reviews (*n* = 255, 11.4%) were the most common types of reference (Figure 2). A total of 470 (21.0%) references were studies from adults.

### Grading of Evidence Supporting the Recommendations on Off-Label Drug Use

**FIGURE 3:**
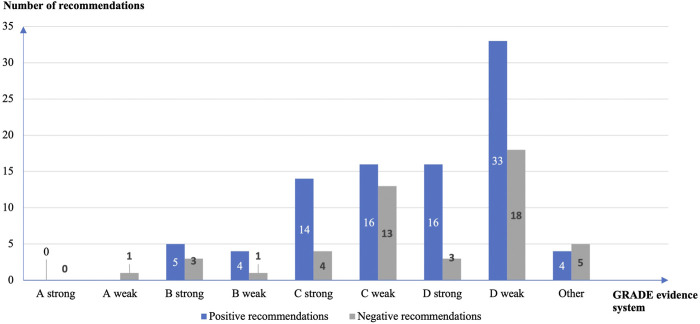
Strength of the off-label drug use recommendations and quality of the supporting evidence according to the GRADE grading system.

The GRADE evidence grading system was used in 140 (23.1%) of the 605 recommendations for off-label use of drugs. The quality of evidence was GRADE level D in 70 (53.4%), GRADE level C in 47 (35.9%), GRADE level B in 13, 9.9%), and GRADE level A in 1 (0.8%) study. There were more weak (*n* = 86, 65.6%) than strong (*n* = 45, 34.4%) recommendations, including 37 (26.4%) strong recommendations supported with GRADE C or D level evidence (Figure 3).

### Information on Off-Label Drug Use

The content and extent of the information reported varied considerably between the recommendations on off-label drug use. The indication was reported in 499 (82.5%), route of administration in 253 (41.8%), dosage in 183 (30.2%), precise dosage in 128 (21.2%), precautions in 175 (28.9%), adverse reactions in 88 (14.5%), course of treatment in 78 (12.9%), contraindications in 30 (5.0%) and other information in 60 (9.9%) recommendations.

### Types of Off-Label Drug Use

Sixty-nine (11.4%) of the 605 assessed recommendations clearly and explicitly stated the use of the drug as off-label. Among the 523 positive recommendations, 255 (48.8%) addressed use for unapproved population, 201 (38.4%) for unapproved indication and 185 (35.4%) for unapproved age, 20 (3.8%) the use of unlicensed drugs, and 15 (2.9%) drug use with unapproved dosage or frequency and 7 (1.3%) with unapproved route of administration.

### Ranking of Specific Off-Label Drugs and Drug Types in the Recommendations

We obtained a total of 941 drug names (652 specific medicines and 289 drug classes) from the 605 off-label drug recommendations. The three most commonly mentioned individual drugs were acetaminophen (*n* = 31, 3.3%), fentanyl (*n* = 28, 3.0%) and ropivacaine (*n* = 24, 2.6%). The three most commonly mentioned drug classes were nonsteroidal anti-inflammatory drugs (NSAIDs; *n* = 46, 4.9%), low molecular weight heparins (LMWHs; *n* = 28, 3.0%), and anticoagulants (*n* = 24, 2.6%) (Supplementary Table S4).

We analyzed detailed recommendations of the top ten off-label drugs (Table 2), of which five drugs were not on the World Health Organization Model List of Essential Medicines for Children. There was fentanyl, ropivacaine, cyclosporine, tramadol, and mycophenolate mofetil. Moreover, there were no references to support the use of infliximab for psoriasis and mycophenolate mofetil for pancreatitis with autoimmune pancreatitis in children, respectively.

**TABLE 2:** The details of the most frequently recommended off-label drugs.

Drug name	WHO EMLc	Types of off-label drug use	Indication	Types of references for recommendations
Acetaminophen	Yes	Unapproved age	Postoperative analgesia	Pediatric case-series
			Pain control after tonsillectomy	Pediatric RCT; systematic review or meta-analysis
			Pain management for acute severe colitis	Pediatric case-series
			Postoperative pain management	Pediatric RCT; pediatric trial; pediatric cohort; pediatric case control study; pediatric case-series; systematic review or meta-analysis; review; pediatric guideline
Fentanyl	No	Unapproved population group	Postoperative pain management	Pediatric RCT; pediatric trial; pediatric case control study; pediatric case-series; systematic review or meta-analysis; review; pediatric guideline
			Pain management for retinopathy of prematurity	Pediatric case-series
Ropivacaine	No	Unapproved population group	Postoperative pain management	Pediatric RCT; pediatric trial; pediatric cohort; pediatric case report; systematic review or meta-analysis; review; pediatric guideline
			Regional anesthesia	Pediatric RCT; pediatric trial; adult trail; pediatric cohort; adult trail; pediatric case control study; adult case control study; pediatric case-series; pediatric case report; adult case report; systematic review or meta-analysis; review; consensus
Cyclosporine	No	Unapproved indication	Ulcerative colitis	Pediatric trial; pediatric cohort; pediatric case-series; adult case series; systematic review or meta-analysis
			Warm autoimmune haemolytic anaemia	Adult RCT; pediatric case-series; review
			Juvenile dermatomyositis	Pediatric case-series; pediatric guideline
			Steroid-resistant nephrotic syndrome	pediatric RCT; pediatric case-series; systematic review or meta-analysis; review
			Psoriasis	pediatric case-series; systematic review or meta-analysis
			Psoriasis	pediatric case-series; pediatric case report; guideline or consensus; expert opinion
			Glomerular diseases	adult RCT
Bupivacaine	Yes	Unapproved indication	Postoperative pain	Pediatric RCT; pediatric trial; pediatric cohort; pediatric case-series; systematic review or meta-analysis; review; pediatric guideline
		Unapproved age unapproved route of administration	Regional anesthesia	Pediatric RCT; adult trail; pediatric cohort; pediatric case control study; adult case control study; pediatric case-series; pediatric case report; adult case report; adult case series; systematic review or meta-analysis; review
Tramadol	No	Unapproved population group	Postoperative pain	Pediatric RCT; pediatric trial; pediatric case-series; cross-sectional study; systematic review or meta-analysis; review; pediatric guideline
Tacrolimus	Yes	Unapproved indication	Ulcerative colitis	Adult RCT; pediatric cohort; pediatric case-series; adult case series; systematic review or meta-analysis
			Ulcerative colitis	Adult RCT; adult trail; adult cohort; pediatric case-series; pediatric case report; systematic review or meta-analysis
			Juvenile dermatomyositis	Systematic review or meta-analysis; guideline or consensus
			Steroid-resistant nephrotic syndrome	Pediatric RCT; systematic review or meta-analysis
			Psoriasis (topical)	Pediatric trial; pediatric case-series; adult case report; systematic review or meta-analysis
			Glomerular diseases during the COVID-19 pandemic	Adult RCT
Infliximab	Yes	Unapproved age	Ulcerative colitis	Pediatric RCT; adult RCT; pediatric cohort; adult cohort; pediatric case control study; pediatric case-series; cross-sectional study; systematic review or meta-analysis; review
			Ulcerative colitis	Pediatric RCT; adult RCT; adult trial; adult cohort; adult case series; cross-sectional study; systematic review or meta-analysis; guideline or consensus
		Unapproved population group	Juvenile idiopathic arthritis	Pediatric RCT
		Unapproved indication	Psoriasis	—
			Psoriasis	Expert opinion
Mycophenolate mofetil	No	Unapproved indication	Autoimmune pancreatitis with a concomitant diagnosis of inflammatory bowel disease	—
			Warm autoimmune haemolytic anaemia	Pediatric case-series; cross-sectional study; systematic review or meta-analysis; review
			Juvenile idiopathic arthritis–associated Uveitis	Adult case control study
			Immunoglobulin A vasculitis	Pediatric RCT; pediatric cohort; adult case control study; pediatric case-series
			Juvenile dermatomyositis	Pediatric case-series
			Steroid-resistant nephrotic syndrome	Pediatric RCT; pediatric cohort
			Glomerular diseases during the COVID-19 pandemic	Pediatric case-series; guideline or consensus
Morphine	Yes	Unapproved population group	Postoperative pain	Pediatric RCT; pediatric trial; pediatric case control study; pediatric case-series; systematic review or meta-analysis; review; pediatric guideline
			Regional anesthesia	Review
			Pain for retinopathy of prematurity	Pediatric case-series
			Needle procedures	Pediatric RCT; cross-sectional study

WHO EMLc: World Health Organization Model List of Essential Medicines for Children (2021).

### Recommendations for Acetaminophen or Nonsteroidal Anti-Inflammatory Drugs (NSAIDs)

Acetaminophen and NSAIDs were the most commonly mentioned drug and drug classes, respectively. Positive recommendations on these drugs provided detailed information on the use of the medication, whereas negative recommendations only addressed the indications that are not suggested (Table 3). The contents of drug information descriptions were diverse among the different diseases, and we could not identify any pattern. The recommendation on NSAIDs for post-tonsillectomy pain control reported the most detailed drug information, including indications, route of administration, dose, precise dosage, adverse effects, and contraindications.

**TABLE 3:** Diseases and information covered by the recommendations on off-label use of nonsteroidal anti-inflammatory drugs (NSAIDs) and acetaminophen.

	Drug	Disease	Indication	Route of administration	Dose	Precise dosage	Course of treatment	Precautions	Adverse reactions	Contraindications
Negative recommendations	Acetaminophen	Acute severe ulcerative colitis	✓							
		Minor procedures	✓							
		Non-systemic polyarthritis, sacroiliitis, and enthesitis	✓				✓			
		Immunoglobulin A vasculitis-associated arthropathy	✓					✓	✓	✓
		Pediatric rheumatic disease	✓					✓		
Positive recommendations	Acetaminophen and NSAIDs	Postoperative pain	✓	✓	✓	✓		✓		
		Pain of pediatrics ulcerative colitis	✓							
	NSAIDs	Symptomatic treatment of arthralgia of psoriasis	✓							
		Painful headache after acute mild traumatic brain injury	✓					✓		
		Otalgia of acute otitis media	✓	✓	✓	✓				
		Pain control after tonsillectomy	✓	✓	✓	✓			✓	✓
		Pain of retinopathy surgery	✓	✓	✓	✓				

## Discussion

### Main Findings

This study identified 605 recommendations on off-label drug use from 66 pediatric guidelines. About 90% of the recommendations did not explicitly designate their recommended drugs as off-label use. Various types of references were cited to support the recommendations, with more than one-fifth being adult studies and 15% of the recommendations lacking references. More than a quarter of the recommendations that were graded using the GRADE evidence system were classified as strong but based on low or very low quality evidence.

### Evidence Supporting the Recommendations on Off-Label Drug Use in Pediatric Guidelines

The majority of clinical decisions are based on the principles of evidence-based medicine (Djulbegovic and Guyatt, 2017). However, in many cases, only low-quality evidence or evidence from adult populations is available to inform the treatment of children. For example, to support off-label pharmacological use in neonates, case reports (Tobin, 2010), expert opinions, and evidence extrapolated from data on other populations are frequently used (Slater et al., 2020). When a new drug is launched, evidence is first usually only available on adults. In the early phases of a public health emergency, only low-level evidence, such as case series, is available (Hudgins et al., 2018; Lagler et al., 2021). Treatment of psychiatric disorders in children is often based on evidence from adults (Sharma et al., 2016), and only case reports are often available for rare diseases (Whitley and Chepke, 2020). As a result, strong recommendations are sometimes generated based on expert consensus (Rosen et al., 2018) and indirect evidence of similar diseases (Liu et al., 2020), as well as based on conclusions from disease features and clinical practice experience. However, the inconsistency between the quality of the evidence and the strength of the recommendation may contradict the critical principles of evidence-based medicine, creating a danger to clinical practice (Yao et al., 2021). As a result, it is critical that recommendations on the off-label use of drugs in pediatric guidelines are based on a reliable methodological pathway or framework (Cheng et al., 2007). However, there is currently no standardized framework for creating recommendations for the use of off-label drugs in children (Casali et al., 2015; Agrawal et al., 2019; Jiang et al., 2020; Stolbach et al., 2020; Remi et al., 2021). In general, the development of clinical guidelines should follow the GRADE Working Group’s “Five Paradigmatic Situations Warranting Strong Recommendation Despite Low or Very Low-Quality Evidence in Effect Estimates,” which has proven to offer a reliable framework (Andrews et al., 2013). This framework, unfortunately, does not resolve the clinical aspects regarding off-label use of drugs in children, and the interpretation and judgments of the GRADE framework may vary between individuals. Furthermore, even if guideline developers strictly adhere to the framework, the subjective nature of expert consensus (Yao et al., 2021), potential conflicts of interest (Stahl, 2013; Joshi et al., 2019), and lack of transparency in many guidelines still present severe risks for clinical practice.

Furthermore, this analysis found that each off-label drug recommendation had an average of 4.3 references. A diverse range of literature types was cited, including books (Cheung et al., 2018), databases (Grover and Avasthi, 2019), internet resources (Leboulanger et al., 2020), instructions (Caffarelli et al., 2019), and scientific letters (Lim et al., 2020). There is a research gap in how to grade evidence from these types of sources in the optimal way (AAP, 2004; Atkins et al., 2004b; Milano, 2015; SIGN, 2017; Blanco-Reina et al., 2017). It is also worth noting that 15% of off-label drug recommendations lacked references, being based on either expert opinions (Menter et al., 2020), or even not reporting the source (Litwin et al., 2018).

### Reporting the Recommendation on Off-Label Drug Use in Pediatric Guidelines

Off-label drug use is presented in the recommendations in diverse ways. Drug-specific guidelines usually report detailed information on the drug, such as pharmacological effects, pharmacokinetic characteristics, dosage or precise dosage, indications, route of administration, co-administration, regimen, contraindications, precautions, adverse reactions, and monitoring (Simm et al., 2018; Zhou P. et al., 2021). In clinical guidelines not focusing on any specific drug, there is however wide variation in how the recommendations on off-label drug use are reported. Indications and methods of administration tend to be reported frequently, whereas information on the dose or accurate dosage is often lacking, and sometimes even the safety indicators are not appropriately addressed. Given the legal (Mayrhofer, 2014) and safety risks (Schrier et al., 2020) associated with pediatric off-label use of drugs (Schrier et al., 2020), it is also critical to clearly describe if a drug in a recommendation is beyond instructions, since the approval of instructions indicates the availability of high-quality evidence-based support. However, less than 15% of the guidelines for children included in this study specifically noted that the recommended use was off-label.

### NSAIDs Used Off-Label

Although many pediatric guidelines suggest NSAIDs or paracetamol for pain, fever or inflammation in children (Turner et al., 2018; Vittinghoff et al., 2018; Mitchell et al., 2019; Ozen et al., 2019; Pirelli et al., 2019; Ringold et al., 2019; Wahezi et al., 2020), they should be taken with caution due to the possibility of adverse reactions such as hypersensitivity reactions (Kidon et al., 2018), renal damage (Gong et al., 2021), and even kidney failure (Gong et al., 2021; Bensman, 2019). However, the reporting of recommendations on NSAIDs in pediatric guidelines is inadequate, which is due to several reasons and may potentially lead to inappropriate drug use. First, pediatric guidelines are usually applicable to children of all ages, but the recommendations do not specify which age groups of children they apply to (Turner et al., 2018). Second, there is a wide range of NSAIDs available, with different therapy ranges and approved ages. Despite this, the recommendations (Ringold et al., 2019) do not declare or clarify which specific NSAIDs are suggested, which might lead to confusion in clinical practice. Finally, as shown in Table 3, the information on NSAIDs stated in the recommendations differs greatly between the guidelines (Turner et al., 2018; Vittinghoff et al., 2018; Ozen et al., 2019; Ringold et al., 2019; Wahezi et al., 2020). There are discrepancies in the content and degree of detail stated in different guidelines when NSAIDs are proposed as off-label drugs, which might be attributable to subjective selection by guideline developers.

### Off-Label Drugs Not on World Health Organization Model List of Essential Medicines for Children

In our research, many off-label drugs recommended by guidelines are not on the World Health Organization Model List of Essential Medicines for Children, but they are evidence-based or rational in many cases. WHO has developed a list of essential medicines mainly based on the safety, efficacy, and cost-effectiveness of medicines over the past 44 years (WHO, 2021a), but the committee has not taken into account feedback from professional societies and organizations that develop clinical guidelines, resulting in the exclusion of medicines with better evidence from the list (Dugani et al., 2018). On the other hand, off-label drugs that are not on the list and recommendations that are not supported by the evidence should be noticed and considered, such as infliximab for psoriasis (Menter et al., 2020), which might have a possible conflict of interest. According to our analysis, more than 10% of off-label drug guidelines involve possible commercial promotion, consistent with the current situation (Abbas et al., 2018).

### Strengths and Limitations

This study is to our knowledge the first systematic analysis of the reporting of off-label drug recommendations in pediatric guidelines and reveals some gaps in reporting. To ensure a correct extraction of data on off-label drug use, experienced clinical pharmacists reviewed and analyzed the recommendations independently, and inconsistencies were discussed with pediatric clinical experts. However, this study also has some limitations. The study only included English-language guidelines from the last 3 years, without searching websites for additional guidelines. Local web pages or local medical organizations’ web pages usually publish guidelines in non-English, which may include more irrational off-label drug recommendations (Meng et al., 2022). To ensure consistency, the FDA prescription instructions were adopted as criteria for categorizing the type of off-label use of drugs, which may not be completely consistent with the drug instructions used in the target setting of the guideline.

## Conclusion

Recommendations on off-label drug use are commonly given in pediatric guidelines, despite the substantial risk of clinical practice being misled. The quality of the evidence supporting the recommendations does not however always match the strength of the recommendations; the recommendations were often based on expert opinion and lacked a declaration of the methodology; and the content and extent of the information reported for recommended off-label drugs varied greatly between the guidelines. Most recommendations involving off-label drug use did not explicitly state that the recommended administration is off-label, which may easily lead to inappropriate off-label use of drugs in children.

## Data Availability

The original contributions presented in the study are included in the article/Supplementary Material, further inquiries can be directed to the corresponding authors.
