# Supplementary material for: Recommendations on Off-Label Drug Use in Pediatric Guidelines
Source: Front Pharmacol. 2022 Jun 9;13:892574. doi: 10.3389/fphar.2022.892574 (PMC9218488; doi:10.3389/fphar.2022.892574)
Supplement: Supplementary file 3 [file Table1.DOCX]

| **Supplementary File 1** | **Search strategy** |
| --- | --- |
|  | **PUBMED search strategy (n=663)** |
| **No.** | search |
| 1 | "adolescent"[MeSH Terms] |
| **2** | "infant"[MeSH Terms] |
| **3** | child[MeSH Terms] |
| **4** | pediatrics[MeSH Terms] |
| **5** | pediatric*[Title/Abstract] OR infant*[Title/Abstract] OR adolescen*[Title/Abstract] OR newborn*[Title/Abstract] OR neonat*[Title/Abstract] OR toddler*[Title/Abstract] OR juvenile[Title/Abstract] |
| **6** | #1 OR #2 OR #3 OR #4 OR #5 |
| **7** | Practice Guidelines as Topic[MeSH Terms] |
| **8** | guideline*[Title] OR recommendation*[Title] |
| **9** | #7 OR #8 |
| **10** | #9 AND Practice Guideline[Publication Type] |
| **11** | #6 AND #10 |
| **12** | #12 AND Fliters：Practice Guideline, Humans, from 2017 – 2019. |
|  |  |
|  | **EMBASE search strategy (n=832)** |
| No. | search |
| #1 | 'adolescent'/exp |
| #2 | 'infant'/exp |
| #3 | 'child'/exp |
| #4 | 'pediatrics'/exp |
| #5 | 'guideline'/exp |
| #6 | pediatric*:ti,ab,kw OR paediatric*:ti,ab,kw OR infant*:ti,ab,kw OR adolescen*:ti,ab,kw OR newborn*:ti,ab,kw OR neonat*:ti,ab,kw OR toddler*:ti,ab,kw OR juvenile:ti,ab,kw |
| #7 | #1 OR #2 OR #3 OR #4 OR #6 |
| #8 | 'guideline*':ti OR 'recommendation*':ti |
| #9 | #5 OR #8 |
| #10 | #7 AND #9 |
| #11 | #10 AND (2017:py OR 2018:py OR 2019:py) AND 'practice guideline'/de AND [EMBASE]/lim NOT ([EMBASE]/lim AND [medline]/lim) |
